# Dynamical Behavior and Conformational Selection Mechanism of the Intrinsically Disordered Sic1 Kinase-Inhibitor Domain

**DOI:** 10.3390/life10070110

**Published:** 2020-07-11

**Authors:** Davide Sala, Ugo Cosentino, Anna Ranaudo, Claudio Greco, Giorgio Moro

**Affiliations:** 1 Dipartimento di Biotecnologie e Bioscienze, Università di Milano-Bicocca, P.zza della Scienza 2, 20126 Milano, Italy; d.sala1388@gmail.com; 2 Dipartimento di Scienze dell’Ambiente e della Terra, Università di Milano-Bicocca, P.zza della Scienza 1, 20126 Milano, Italy; ugo.cosentino@unimib.it (U.C.); a.ranaudo@campus.unimib.it (A.R.)

**Keywords:** Sic1, IDPs, MD simulations, force field, WHIM descriptors, conformational selection, molecular shape, conformational ensemble

## Abstract

Intrinsically Disordered Peptides and Proteins (IDPs) in solution can span a broad range of conformations that often are hard to characterize by both experimental and computational methods. However, obtaining a significant representation of the conformational space is important to understand mechanisms underlying protein functions such as partner recognition. In this work, we investigated the behavior of the Sic1 Kinase-Inhibitor Domain (KID) in solution by Molecular Dynamics (MD) simulations. Our results point out that application of common descriptors of molecular shape such as Solvent Accessible Surface (SAS) area can lead to misleading outcomes. Instead, more appropriate molecular descriptors can be used to define 3D structures. In particular, we exploited Weighted Holistic Invariant Molecular (WHIM) descriptors to get a coarse-grained but accurate definition of the variegated Sic1 KID conformational ensemble. We found that Sic1 is able to form a variable amount of folded structures even in absence of partners. Among them, there were some conformations very close to the structure that Sic1 is supposed to assume in the binding with its physiological complexes. Therefore, our results support the hypothesis that this protein relies on the conformational selection mechanism to recognize the correct molecular partners.

## 1. Introduction

Intrinsically Disordered Peptides and Proteins (IDPs) are biomolecules featuring a high degree of structural diversity, which is tightly related to their function [1,2]. The main feature of IDPs is the structural flexibility that allows them to assume a wide range of conformations [3,4,5]. However, some of them are able to visit structured and compact states in solution, then fold into a specific structure when they bind to either a target molecule or cofactor [6,7]. IDPs are very common in nature, 30% of eukaryotic proteins are predicted to be partially or wholly disordered [8]; moreover, up to 70% of proteins involved in signal transduction pathways may contain intrinsically disordered regions [9]. Two of the proposed advantages of IDPs are adaptability of their shapes to different binding targets and the capability to do a fast binding process due to their long capture radius, which is called the fly-casting mechanism [10].

Despite their highly flexible structure combined with preformed dynamic binding elements, IDPs are usually able to escape unwanted interactions with non-native partners via the so-called functional misfolding [11]. According to this model, the preformed secondary structure elements are dynamically involved in a set of intramolecular electrostatic and hydrophobic interactions inside a less-interactive cage, where they are dynamically excluded from the environment and therefore might escape unwanted interactions with the non-native binding partners [12,13,14]. 

IDPs play an important role in the regulation of the cell cycle [15]. The cyclin-dependent-kinase inhibitor (CKIs) Sic1 from *Saccharomyces cerevisiae* is a key protein for the control of entrance into S phase of the yeast cycle [16]. The transition from G1 to S phase during the mitotic cell cycle is accomplished through the formation of complexes between the kinase Cdk1 and the cyclins Clb5 or Clb6 [17]. The kinase activity of the complex is inhibited by Sic1 binding until a multiple phosphorylation on Sic1 N-terminal, performed by Cdk1/Cln1,2, triggers its ubiquitin-dependent proteolysis [18]. Sic1 is also involved in other cellular processes, such as cell size regulation, exit from mitosis, and signaling in the stress-response pathways [19,20].

Sic1 is a 284-residue-long protein, it is disordered along the whole sequence, nevertheless it is able to form secondary and tertiary structures in solution [21]. The last 70 residues of the protein form the minimal fragment with inhibitory activity [22,23], the so-called Kinase-Inhibitor Domain (KID) domain, which is functionally and structurally related to the inhibitory domain of mammalian p27^Kip1^ [24]. The availability of p27-Cdk2-cyclin A crystal structure allowed to predict the yeast ternary complex based on the template of the mammalian one [25].

Sic1 structural properties in solution were studied experimentally and in silico through a set of biophysical methods such as Electrospray Ionisation Mass Spectrometry (ESI-MS), Circular Dichroism (CD), limited proteolysis [26], and by Molecular Dynamics (MD) simulations [27]. The results indicated that Sic1 has properties typical of pre-molten and molten globular proteins. Moreover, the KID fragment—even though disordered—is the Sic1 most structured region, showing both folded secondary structures and a certain propensity to form partially collapsed conformations. In this regard, all atoms molecular dynamics is a useful technique to study conformational properties of proteins. However, it is still difficult to define tertiary structures inside a wide range of conformations that characterize IDPs. 

In this work, atomistic MD simulations in explicit solvent were carried out in order to explore structural properties of the Sic1 KID in solution. We exploited two force fields respectively developed to sample folded (charmm22*) and disordered conformations (Amber ff03ws) in the native state [28,29], thus being somehow complementary with each other. To analyze MD-based conformational ensembles of IDPs, a number of metrics such as radius of gyration, secondary structures content, RMSD, number of hydrophobic contacts, as well as collective variables (CVs) in enhanced sampling simulations have been successfully applied [30,31,32]. However, to obtain a satisfactory picture of a highly heterogeneous conformational space it is often necessary to use multiple metrics/CVs that are not always trivial to select and may rise the computational cost remarkably. Here, the Weighted Holistic Invariant Molecular (WHIM) descriptors were used to analyze conformational ensembles. The WHIM descriptors are geometrical descriptors based on statistical indices calculated on the projections of the atoms along principal components [33]. Indeed, they were developed to describe 3D structural properties such as molecular size, shape, symmetry, and atom distribution and they have been extensively used in QSAR applications [34,35,36]. 

In good agreement with ESI-MS spectra, by using the WHIM descriptors we were able to identify two main populations. Moreover, the peptide in solution is able to assume structures predisposed to bind Cdk/cyclin complex, even in the absence of molecular partners.

## 2. Methods 

This work is based on MD simulations of the Sic1 KID inhibitory domain (residues 215–284). All the simulations were performed using GROMACS [37]. The structure of the peptide was built from scratch, without any structural template. The peptide was capped on N- and C-termini with the ACE and NME caps, respectively. All the histidine residues were kept neutral and protonated on the N-*δ* tautomer. Three fragments were modeled to share a common completely linear 3D structure but with a different type of secondary structure along the whole sequence. As a result, the three linear fragments feature a secondary structure based on random coil, α-helix, and β-strand motif, respectively. However, the use of any of these extremely elongated conformations as starting point for an MD simulation in explicit water would have forced the use of a very large simulation box full of water molecules, resulting in a very poor conformational sampling performance. To avoid this, we first set up three short simulations in implicit solvent aimed at generating globular or partially globular conformations to be used as starting models for the explicit solvent simulations. Each system was minimized with the steepest descent algorithm followed by conjugate gradient. In order to boost the conformational sampling, the temperature of the systems was set to 400 K and controlled with the velocity rescaling algorithm and a relaxation time of 0.1 ps. The GBSA approach for implicit solvent simulations was used and the electrostatic interactions were treated with the OBC model using a cutoff of 0.8 nm [38]. The timestep was set to 2 fs and all bond lengths were constrained using the LINCS algorithm. Initial atom velocities were taken from a Maxwellian distribution at 400 K. The structures in output from the implicit solvent runs were taken as starting conformations for the runs in explicit solvent.

In explicit solvent we performed six runs of 100 ns each, three of them using the Amber ff03ws force field in combination with the TIP4P/2005 water model and three using charmm22* force field in combination with TIP3P water model [28,29].

With the latter force field, the systems were solvated in a dodecahedral box with Periodic Boundary Conditions (PBC). The box size was chosen so that the minimal distance between the atoms of the peptide and the box walls was 1.2 nm. Each system was kept electrically neutral. After a brief minimization with the steepest descent method, the solvent was equilibrated with a MD run of 50 ps applying positional restraints to the peptide with a 1000 kJ mol^−1^ force constant in an NVT ensemble followed by a 100 ps run with a constant pressure of 1 bar. The production runs were performed in a NPT ensemble where protein and non-protein atoms were each coupled to two thermal baths at 400 K with the V-rescale algorithm and constant coupling of 0.1 ps. Pressure was kept at 1 bar using the Berendsen barostat every 1 ps. Cut-off of 0.8 nm was used for Lennard–Jones and Coulomb interactions, which were calculated using the Particle-Mesh Ewald (PME) method, 0.12 nm grid-spacing and a fourth-order polynomial interpolation. The integration step was set to 2 fs, initial atom velocities were taken from a Maxwellian distribution at 400 K. For each model we run 100 ns of production run saving atomic coordinates every 1 ps. 

With the Amber ff03ws force field, a bigger dodecahedral box was built with a distance of 2 nm between the solute atoms and the box walls. The peptide was soaked in the 4-atoms TIP4P2005 water model and neutralized. The resulting systems were minimized and equilibrated as done for the charmm22* runs but with a cutoff of 1.2 nm for electrostatic interactions. For each model, a simulation of 100 ns with an integration step of 2 fs was carried out. The frames in which the protein touches its image through a PBC wall were removed from the trajectories. In addition to the simulations at 400 K, for each model a simulation of 100 ns at 300 K was carried out to check how much temperature affects conformational sampling. 

The secondary structure for each conformation was computed with the Define Secondary Structure of Proteins (DSSP) algorithm [39,40].

The Solvent Accessible Surface (SAS) area was calculated using a 0.14 nm probe. 

The Principal Component Analysis (PCA) to derive the Essential Dynamics (ED) space was performed on C⍺ atoms.

The WHIM descriptors were developed to provide a tridimensional description of molecular structure in terms of size and shape [33]. When dealing with proteins, the tertiary structure can be characterized on the basis of cartesian coordinates of backbone atoms. However, extracting relevant information from the huge amount of conformations that usually are sampled by MD simulations is not trivial. A common way to do so is through methods for dimensionality reduction of molecular structures. Among them, WHIM descriptors can be derived by computing the PCA on the cartesian coordinates. In this work the PCA was performed on backbone atoms (C, C⍺, O, N, H). Then, by projecting atomic coordinates along the three principal components (PCs) of the new rotated space, we calculated all the 13 WHIM descriptors. The three main descriptors (*λ*_1_, *λ*_2_, *λ*_3_) are the eigenvalues of the PCA, and are related to molecular size. From these three size descriptors, two shape descriptors (ϑ_1_, ϑ_2_) Equation (1), three global size descriptors (*T*, *A*, *V*) Equations (2)–(4), and one global shape descriptor (*K*) Equation (5) can be derived as reported in the following equations.

(1) $$\vartheta_{m}=\frac{\lambda_{m}}{\sum_{m}\lambda_{m}}\;m=1,2$$

(2) $$T=\lambda_{1}+\lambda_{2}+\lambda_{3}$$

(3) $$A=\lambda_{1}\lambda_{2}+\lambda_{1}\lambda_{3}+\lambda_{2}\lambda_{3}$$

(4) $$V=\overset{3}{\underset{m=1}{\prod }}(1+\lambda_{m})-1=T+A+\lambda_{1}\lambda_{2}\lambda_{3}$$

(5) $$K=\frac{\sum_{m}|\frac{\lambda_{m}}{\sum_{m}\lambda_{m}}-\frac{1}{3}|}{\frac{4}{3}}$$

Three further density descriptors (*η*_1_, *η*_2_, *η*_3_) can be derived from the inverse of the kurtosis to define the global density index *D* Equation (6):

(6) $$D=\left(\eta_{1}+\eta_{2}+\eta_{3}\right)/\;3$$

The index K rigorously defines geometric shapes, in the range 0–1, where 0 represents a sphere and 1 a straight line. Thus, this index K is particularly relevant to the present work because it allows to distinguish between compact and extended structures, referring to their shape. The same goal of defining molecular shape through a metrics can be achieved by computing SAS area that measures the molecular accessibility to water molecules. However, the index K defines the conformational shape from atomic distributions, whereas SAS area defines molecular shape indirectly by molecular accessibility measurements that can be affected by other structural features such as the presence of a significant number of accessible cavities. Therefore, by using the shape-index K one has a precise and rigorous geometrical measurement of the molecule, whereas by calculating SAS area one may obtain cavity-biased values thus overestimating extended structures.

The distributions of K and SAS values in the conformational ensembles were deconvoluted with the minimum number of gaussian curves which provides a fitting R^2^ value higher than 0.990.

Given two reaction coordinates q and α the probability to find a system in a specific state is given by $e^{-G\frac{\left(q,\alpha \right)}{kT}}$ where G(q,α) is the Gibbs of the state. The Free Energy Landscape (FEL) can be derived from G(q,α)=−kTln[P(q,α)] where *k* is the Boltzmann constant, *T* the temperature of the simulation, and P(q,α) is an estimation of the probability density function. 

In this work all the cluster analyses were carried out with the GROMOS algorithm [41]. The RMSD calculations were performed on the backbone atoms.

## 3. Results

In this study we investigated by MD simulations the conformational ensemble of Sic1 KID fragment consisting of 70 residues all predicted to be disordered is solution. First, we built three models of the peptide with a completely linear shape. Each model has a different secondary structure along the whole sequence consisting of a random coil, α-helix, and β-strand motif, respectively. For each model two simulations of 100 ns were performed using two different force fields, the Amber ff03ws force field and the charmm22* force field [28,29]. The ff03ws and charmm22* force fields were respectively parametrized on disordered states and on globular/folded proteins. All the 100 ns runs were carried out at 400 K. Such high temperature allows one to explore a consistent portion of the conformational space in a short simulation time. The conformational ensemble collected for each force field was analyzed as a unique dataset. 

In a previous work, the secondary structure of the KID fragment in solution was assessed experimentally by Fourier Transform Infrared (FT-IR) spectroscopy [26]. The peptide was found prevalently random-coil, confirming its disordered nature, but other structured components were also identified and quantified as reported in Table 1. The DSSP analysis of the two conformational ensembles evidences that the peptide tends to sample mostly a disordered state with some propensity to exhibit structured regions. A similar amount of turn structures appears to be present in our simulations, as compared to FT-IR data. However, there is about half of the helix structures in our trajectories and even less β-strand structures, having the latter experimental results as a reference. Between the force fields, the charmm22* ensemble has four times more β-strand structures than ff03ws.

**Figure 1:**
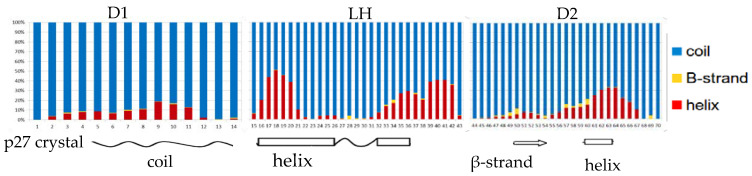
DSSP (Define Secondary Structure of Proteins) analysis of the charmm22* conformational sampling. The Sic1 KID is split in three functional subdomains in accord to the secondary structure of p27 KID bound to Cdk2/cyclin A.

The crystal structure of the homologue p27 human protein bound to Cdk2-cyclin A suggested the presence of three subdomains in the KID fragment associated to a specific secondary structure [25]. The disordered D1 domain is located in the first 10 residues and binds the RxL motif of cyclin A, the D2 domain in the last 30 residues is partially structured and binds CdK2. Finally, the LH domain is a dynamical helix separating the subdomain D1 and D2. Considering that Sic1 KID is able to bind and inhibit the same complex of p27 [24], we measured the secondary structure per-residue by DSSP and compared the results with respect to the secondary structure found in the crystal structure of the p27 homologue KID (Figure 1). As the values observed in the two conformational ensembles were very similar, we reported in the plot only the charmm22* fractions per-residue. The plot shows an analogy between the propensity to form secondary structures along the sequence of Sic1 in solution and p27 in the bound state, in particular for the LH domain where, although less extended, a long helix appears clearly in correspondence of the p27 helix. The D2 domain in the crystal structure has a β-strand formed by residues 50–54 and a short helix between residues 60 and 63. In addition, it was previously observed by NMR that p27 KID in solution can turn the short β-strand in a helical structure [42]. In accord to this, among the folded structures our simulations sampled mostly a helix and in part a β-strand in that region.

**Figure 2:**
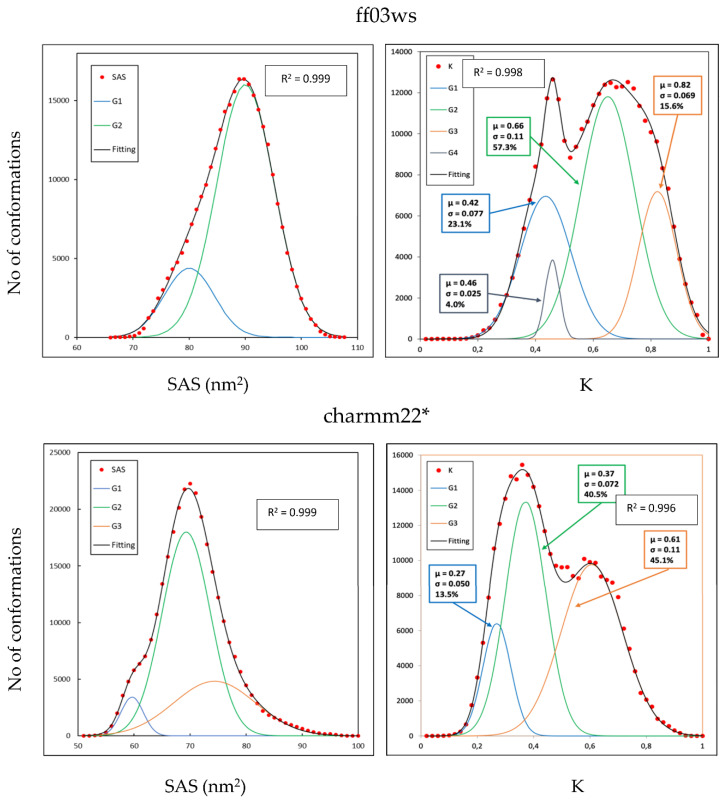
Frequency distribution of Solvent Accessible Surface (SAS) area and Weighted Holistic Invariant Molecular (WHIM) descriptor K.

The tertiary structure of the Sic1 KID fragment was estimated from ESI-MS data showing a bimodal distribution in which 78% of the structures were found in the extended state with an average SAS area of 88.76 nm^2^ against a 22% observed in the compact state with an average SAS area of 59.78 nm^2^ [26]. Here, for both the conformational ensembles we computed the SAS area and the WHIM descriptors to obtain a coarse-grained representation of the 3D structures in our conformational sampling (Figure 2). The computed SAS area of the ff03ws ensemble revealed a substantially unimodal distribution in which the biggest population represents 81.0% of the total and has a mean SAS area of 89.83 nm^2^, therefore close to the experimental values for elongated conformations. However, a second small population emerged, representing 19% of the sampling, that includes conformations with a mean value of 79.81 nm^2^, much higher than the experimental value for the compact state. 

Among the WHIM descriptors, the shape-index K gives a global representation in which a value of 0 and 1 represents an ideally spherical or linear molecule, respectively. The calculated K values of the charmm22* ensemble evidenced a bimodal distribution composed by four populations, the largest one (57.3% of the total) with a mean K value of 0.66 corresponding to partially extended conformations. The other three populations have a mean K values of 0.42, 0.46, and 0.82 representing partially compact states in the first two cases (23.1% and 4.0% of the total, respectively) and extended structures in the last one (15.6% of the total).

In line with the SAS distribution of the ff03ws ensemble, also the charmm22* ensemble has a substantial unimodal SAS distribution in which the largest population has a mean value of 70 nm^2^, halfway between the values experimentally observed for compact and extended states. Two other smaller populations emerged representing compact and extended conformations. Therefore, differently from what observed in the ff03ws ensemble, here there are also SAS values experimentally observed for compact states. The shape-index K distribution shows, as in the charmm22* case, a bimodal distribution composed by three main populations with a mean value of 0.27, 0.37, and 0.61 (13.5%, 40.5%, and 45.1% of the total sampling, respectively) that can be assigned to compact, partially compact, and partially extended conformations. Overall, in this conformational ensemble partially extended and compact conformations seem equally distributed.

**Figure 3:**
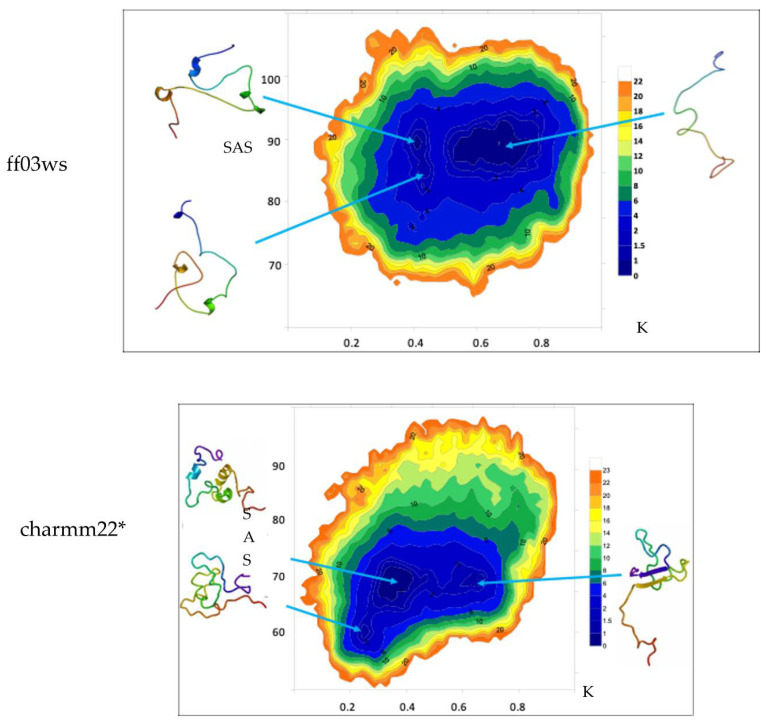
Free Energy Landscape (FEL) (kJ mol^−1^) in the K and SAS (nm^2^) space. Conformations in each basin are shown.

The free energy landscape (FEL) calculated in the K/SAS space is reported for both the conformational ensembles (Figure 3). 

The ff03ws FEL displays three populated basins. The extraction of a representative structure from each basin by cluster analysis shows that the largest population includes conformations that feature high solvent accessibility and extended shape. Instead, the other two smaller populations contain conformations with similar K values but different solvent accessibility. However, the representative structures look pretty similar with a shape at halfway between globular and extended. The same analysis on the charmm22* ensemble shows two main populations with similar SAS area but two different K values. Between them, the population with a higher K value includes conformations that appear more extended than those in the lower K value region that instead encompasses rather compact states. Noteworthy, a third smaller population with a K value similar to the one characterizing the population with compact structures, but differing in SAS area, features compact states as well. As a result, here the two populations distributed along the K axis correspond to conformations differing in terms of shape, whereas the two populations along SAS axis include similar shape conformations.

**Figure 4:**
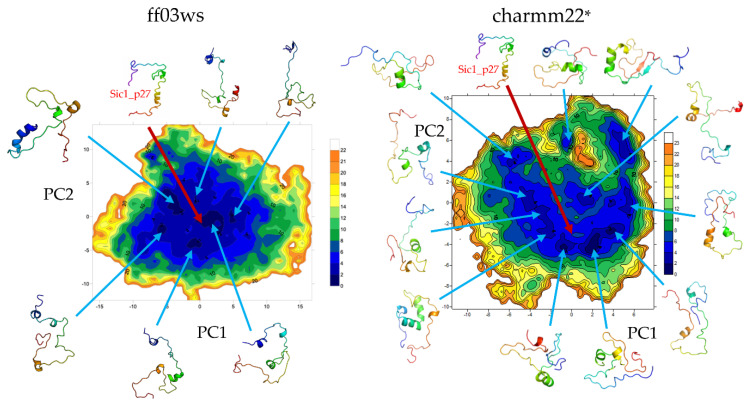
FEL (kJ mol^−1^) on the PC1/PC2 Essential Dynamics (ED) space. Conformations in each minimum are shown. The projected Sic1_p27 structure is shown with a red arrow.

The essential dynamics (ED) space was obtained by performing principal components analysis (PCA) on the ff03ws and charmm22* conformational sampling. The sum of the resulting first two PCs represent 40% and 33% of the ff03ws and charmm22* overall motions, respectively. The ff03ws PC1/PC2 FEL shows a number of global minima separated by low energy barriers (Figure 4). The representative structures of these minima cover a range of different conformations rather elongated. In order to verify whether Sic1 in solution can sample conformations close to the bound state, we modelled the Sic1 KID on the crystal structure of the homologue p27 KID bound to the Cdk/cyclin complex. The resulting Sic1 bound-like conformation (hereafter called Sic1_p27) was then projected on the PC1/PC2 FEL and is located on the edge of the minimum populated by conformations that have a similar shape.

In the charmm22* ED space there is a significant number of minima that include different conformations spanning from compact to partially extended. The projection of the Sic1_p27 structure is located in a local minimum with relatively low free-energy (<8 kJ mol^−1^).

**Figure 5:**
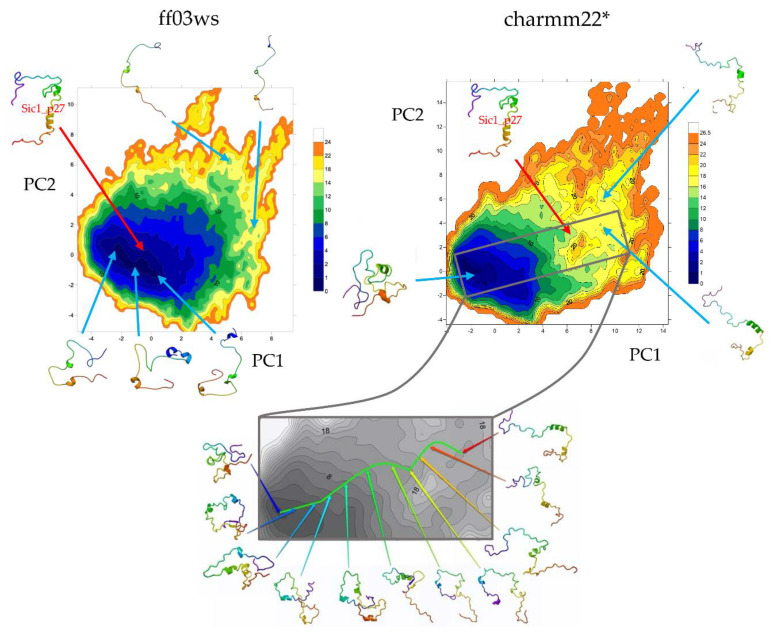
FEL on the PC1/PC2 WHIM descriptors space. Conformations corresponding to cluster centroids of each minimum are shown. Projected in the gray box are shown the conformations extracted along the pathway of minimal gradients that connects the extended structures from the compact state structures of the global minimum.

The PCA was then performed on the two conformational ensembles described by the autoscaled WHIM descriptors. The sum of the first two components represents 61% of the WHIM space in both the ff03ws and charmm22* ensembles. PC1 prevalently describes molecular shape, while PC2 molecular size. This coarse-grained representation allows to identify in a wide minimum the most accessible conformations that suggestively, in the ff03ws ensemble, can be either partially compact or partially extended (Figure 5). Despite being in the same global minimum, more globular conformations are located on the left of the minimum, whereas more extended structures on the right. Noteworthy, very extended conformations are located in high-energy minima having high PC1 or PC2 coordinates. The charmm22* FEL shows a global minimum encompassing with a globular shape. Instead, extended conformations are located in scattered local minima with higher free energy. Then, by computing the free-energy gradients on the FEL we identified a pathway of minimal gradients that connects the global minimum encompassing compact and “globular” structures with a local minimum that includes extended conformations. Therefore, the conformations extracted along this pathway represent transition states connecting compact state conformations to extended structures. Notably, the projection of the Sic1_p27 structure in this space is located along the pathway of minimal gradients, suggesting that it might be accessible also in solution. 

**Figure 6:**
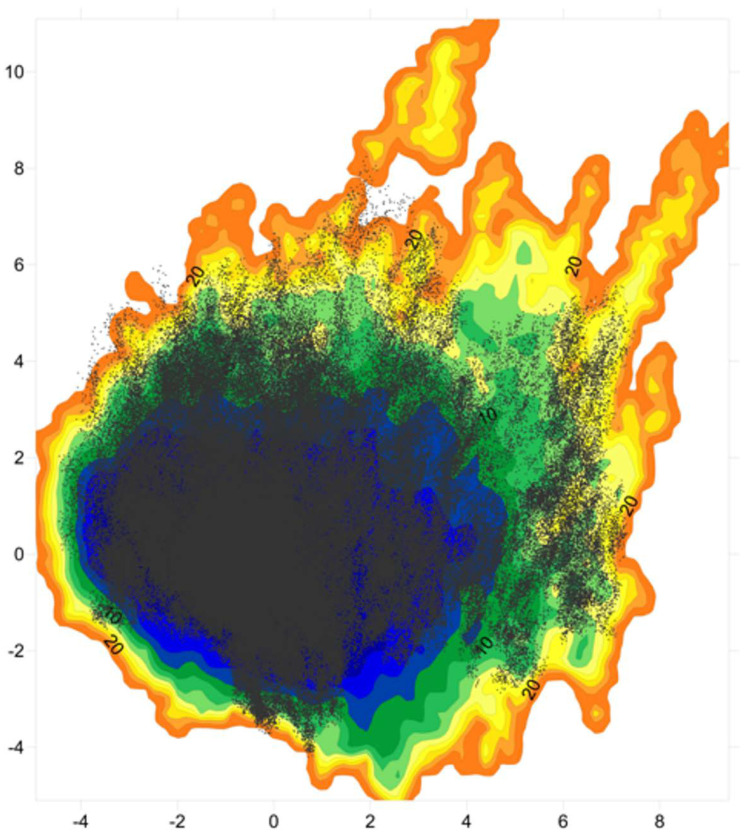
Projection of the 300 K conformational ensemble on the PC1/PC2 WHIM descriptors space calculated on the conformational ensemble at 400 K.

To analyze whether the high temperature improperly affects the Sic1 sampling we carried out three simulations with the ff03ws force field at 300 K. The resulting WHIM scores were then projected on the WHIM PC1/PC2 space calculated at 400 K (Figure 6). The structures sampled at 300 K cover most of the space explored at 400 K showing that compact and partially compact conformations are accessible also at 300 K. Among them, a significant number of conformations sampled the same basin of the Sic1_p27 projection. Extended conformations were also found, although with a lower frequency with respect to the 400 K conformational ensemble.

**Figure 7:**
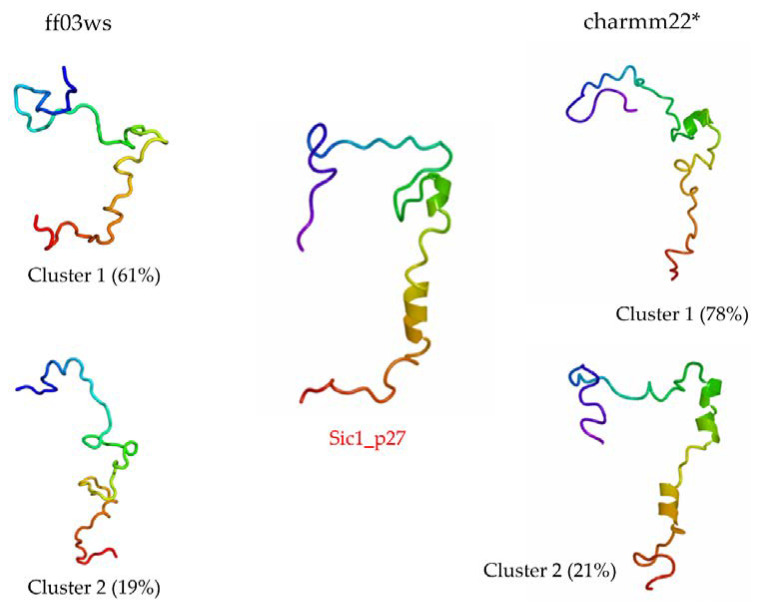
Cluster analysis performed on the structures with a RMSD below 1 nm from Sic1_p27. The centroid of the first two clusters are shown. In the middle the Sic1_p27 structure.

The RMSD with respect to Sic1_p27 was computed to estimate the number of sampled conformations that feature a shape close to the bound state. Then, a cluster analysis was carried out including only the structures below 1 nm from Sic1_p27. In the ff03ws ensemble, 1119 structures were below 1 nm from Sic1_p27. The first two clusters out of 13, respectively, represent 60.8% and 19.5% of the pool, and have the closest structure to the corresponding centroid, i.e., centrotype, quite similar to Sic1_p27 (Figure 7). The RMSD calculation on the charmm22* ensemble resulted in 846 structures grouped in four clusters, the first two of which represent 78% and 21% of the total and have centrotype structures impressively similar to Sic1_p27.

## 4. Discussion

In summary, with both the Amber ff03ws and the charmm22* force fields, the Sic1 KID in solution sampled an excessive amount of unfolded secondary structures with respect to the values observed experimentally [26]. Despite a similar amount of turn structures, the sampled conformations lack half of helical content and almost completely β-strands. Between the conformational ensembles, the charmm22* force field sampled few more folded secondary structures than the Amber ff03ws. It is noteworthy that folded secondary structures, in particular helices, were prevalently observed in the same residues previously identified as structured in the homologue p27 KID in solution or bound to the Cdk2/cyclin A complex [25,42].

The picture that emerges from literature for the Sic1 KID in solution depicts a very flexible peptide displaying transient secondary and tertiary structures [21,26,27]. ESI-MS measurements revealed the Sic1 KID sampling approximatively 30% globular/compact states and 70% extended/accessible states. In our simulations, the distribution of 3D structures in the conformational ensemble was assessed by computing SAS area and WHIM descriptors. Among the WHIM descriptors, the shape-index K provides a global representation of the molecular shape [33]. The SAS area distribution of the ff03ws ensemble indicates most of the conformations as highly solvent accessible. Instead, SAS area values of the charmm22* ensemble show a roughly unimodal distribution with a mean value halfway between the values experimentally associated to globular and extended conformations. Although in both cases the SAS area distribution suggested that compact and globular states are rare, by measuring the shape-index K we identified a shape distribution in which different populations emerge. In the ff03ws ensemble we found populations with K values compatible with partially “globular”, partially extended and extended conformations. In the charmm22* ensemble the shape-index K displayed a distribution in which compact and extended conformations are almost equally distributed. According to this, the corresponding FEL in the K/SAS space of the two ensembles—see Figure 3—exhibits two main populations with similar SAS area but different K values containing structures characterized by different shape. Conversely, the two populations with similar K values but different SAS area include structures with very similar shape. These results raise questions on the reliability of SAS area as shape descriptor.

The FEL on the first two PCs of the ED space included about 40% of the variance in both the conformational ensembles. In the ff03ws FEL the minima were mainly populated by elongated conformations. One of them included conformations similar to the structure of Sic1 modeled on the p27 KID in the bound state (Sic1_p27) that indeed had a projection in proximity of that minimum. In the charmm22* FEL the minima were instead mainly populated by globular or partially globular states. According to this, the Sic1_p27 projection was located in a local minimum.

The FEL on the first two PCs of the WHIM space was much representative describing about 60% of the variance in both the ensembles. The ff03ws FEL shows in a unique large global minimum a number of conformations ranging from partially globular to extended, that therefore might be both accessible. On the contrary, the charmm22* FEL turned out to have a global minimum including only globular conformations, while extended conformations were located in diverse and scattered local minima. By computing the pathway along the minimal gradients connecting the global minimum with a local minimum in the nearby, a number of conformations were extracted showing putative transition states between compact and extended conformations. Interestingly, the projection of the Sic1_p27 structure along the pathway of minimal gradients suggests that Sic1 might be able to sample conformations close to the bound state even in absence of molecular partners.

The simulations at 300 K with the ff03ws force field yielded a range of conformations covering a large portion of the 400 K WHIM space. Moreover, structures similar to the bound state might be accessible also at physiological temperature. Although with a lesser frequency, extended conformations were also present at 300 K.

A further confirmation in this direction comes from the cluster analysis performed on the structures with a RMSD value below 1 nm from Sic1_p27. The centrotypes of the first two most populated clusters show structures that in the case of the ff03ws ensemble were quite close to Sic1_p27 or, in case of the charmm22* ensemble, were impressively similar to Sic1_p27.

A final remark concerns the two force fields adopted in this study, namely the Amber ff03ws and the charmm22*. They were developed to sample as native state unfolded/disordered and globular/folded states, respectively. The highly dynamical behavior of IDPs populates global minima with a wide range of different conformations separated by metastable partially structured intermediates that can become significantly populated especially at high temperature [43]. In the case of Sic1 KID, the conformational ensembles determined by both force fields sampled different type of conformations that turned out to be separated by metastable partially folded states among which we found conformations close to the bound state. Therefore, both the Amber ff03ws and charmm22* force fields sampled a satisfactory amount of relevant conformations, even though with a different frequency.

## 5. Conclusions

WHIM descriptors, and in particular the global shape-index K descriptor, have demonstrated to be more robust in describing the conformational ensemble in terms of shape than a popular descriptor such as SAS area. The latter actually can suffer strong uncorrelation with molecular shape.

Our simulations confirmed that Sic1 KID can assume partially folded secondary structures and can span different conformations ranging from compact to elongated, a behavior typical of molten globule and pre-molten globule IDPs. Among the accessible conformations, compact and globular states might help the protein to escape unwanted interactions with non-native partners, as proposed by the functional misfolding model. At the same time, the presence of elongated structures very close to the bound state tempts us to hypothesize that the so-called conformational selection mechanism—through which the correct molecular interactors are recognized—might be crucial for the Sic1 KID, as previously reported for p27 KID [44]. Although more experimental evidence is required, the observed intrinsic dynamical exchange between extended and compact conformations seems to be functional for the interaction with the Sic1 KID partner.

## Figures and Tables

**Table 1:** Fraction of secondary structures.

	Coil	Bend	Turn	Helix	β-Strand
FT-IR [26]	0.40	-	0.15	0.30	0.15
ff03ws	0.53	0.20	0.13	0.13	0.01
charmm22*	0.43	0.21	0.15	0.17	0.04
